# Pediatric adenovirus infections: 10-year clinical spectrum and predictors of severe disease with emphasis on comorbidities and coinfections

**DOI:** 10.1007/s00431-026-06952-0

**Published:** 2026-04-24

**Authors:** Kubra Aykac, Aylin Irmak Kuruc, Osman Oguz Demir, Emine Dilara Karatas, Fatma Ugur, Gulrukh Tuychiboeva, Ali Bulent Cengiz, Malik Aydin, Yasemin Ozsurekci

**Affiliations:** 1 Department of Pediatrics Infectious Diseases, Hacettepe University Faculty of Medicine, 06100 Ankara, Turkey; 2 Department of Pediatrics, Hacettepe University of Medicine, 06100 Ankara, Turkey; 3 Hacettepe University, School of Medicine, 06100 Ankara, Turkey; 4 Laboratory of Translational Medicine and Pediatric Infectious Diseases, Center for Biomedical Education and Research (ZBAF), Faculty of Health, Department of Human Medicine, Witten/Herdecke University, Stockumerstr. 10, 58453 Witten, Germany; 5 Chair of Pediatrics, Vestische Kinder- und Jugendklinik Datteln, Witten/Herdecke University, 45711 Datteln, Germany

**Keywords:** Human adenovirus, Pediatric infection, Hospitalization, Comorbidity, Coinfection

## Abstract

Human adenoviruses (HAdVs) may cause and are responsible for a broad range of pediatric illnesses. Although several cases are self-limiting, certain children may develop complications requiring pediatric intensive care (PICU). To address the role of HAdVs in children, we aimed to characterize the 10-year clinical spectrum of pediatric HAdVs infections and to identify factors associated with hospitalization and severe disease. This retrospective observational cohort study analyzed the clinical course of children (< 18 years) with laboratory-confirmed HAdV infection episodes treated at a tertiary pediatric center between 2014 and 2024. Demographic features, comorbidities, laboratory results, respiratory support requirements, PICU, and clinical outcomes were collected. The severe outcome was defined as a composite endpoint including PICU admission, invasive mechanical ventilation, and/or 30-day mortality. A total of 877 HAdV infection episodes were included, whereas 276 (31.5%) of the patients were hospitalized. Among hospitalized patients, 32.2% required respiratory support, 18.8% required PICU care, and the 30-day mortality was 2.5%. Overall, 104 children (11.9%) developed severe disease. Underlying medical conditions were significantly more frequent among children with severe disease, while those without underlying disease were more common in the non-severe group (69.8% vs. 22.1%; *p* < 0.001). Viral coinfections were detected in 35.8% of patients but were not associated with increased hospitalization or severity. In multivariable analysis, male sex (OR 2.30, 95% CI 1.23–4.29; *p* = 0.009), neurologic disease (OR 2.12, 95% CI 1.01–4.42; *p* = 0.045), cardiac disease (OR 3.27, 95% CI 1.49–7.17; *p* = 0.003), and hematopoietic stem cell transplantation (HSCT) (OR 4.32, 95% CI 1.36–13.7; *p* = 0.013) were independently associated with severe outcome.

*Conclusion*: Human adenovirus infection represents a notable clinical burden in children, with a considerable proportion requiring hospitalization and developing severe disease. Underlying neurologic and cardiac diseases, as well as HSCT, were key risk factors for severe outcome, whereas viral coinfections were common but not associated with worse clinical outcome.

**Supplementary Information:**

The online version contains supplementary material available at 10.1007/s00431-026-06952-0.

**Table:** 

**What is Known?** • *Human adenovirus (HAdV) infections are common in children and usually self-limiting but may lead to severe disease.* • *Underlying medical conditions and immunosuppression have been associated with worse clinical outcomes in pediatric HAdV infections.*
**What is New?** • *Underlying neurologic disease, cardiac disease, and HSCT were identified as independent predictors of severe outcome.* • *The findings highlight that host-related factors rather than viral coinfection are the main aspects of disease severity.*

## Introduction

Human adenovirus (HAdV) infection is a major cause of acute illness in children worldwide, accounting for a significant proportion of viral infections. Besides respiratory tract infections, HAdVs are also associated with conjunctivitis, gastrointestinal infections, and different extrapulmonary manifestations, including hepatitis, myocarditis, and central nervous system involvement. Although most infections are self-limiting, severe and life-threatening disease may occur [1–4].

The clinical course of HAdV infection is highly variable, ranging from mild outpatient illness to severe pneumonia requiring intensive care and respiratory support [5]. Several studies have identified age, disease comorbidities, and high viral load as potential contributors to a severe disease course. However, the risk factors of hospitalization and severe outcomes in children still remain incompletely defined [4, 6]. Furthermore, the role of viral coinfections on disease severity has shown inconsistent results across different pediatric populations [7, 8].

Despite the recognized clinical importance of HAdV infections, long-term epidemiologic data describing their burden and severity patterns in children are limited, in particular from middle-income countries [9, 10] The understanding of seasonal or temporal trends, risk profiles, and clinical predictors of severe disease is essential for early recognition, triage decisions, and supportive care strategies.

To address this, we aimed to characterize the 10-year epidemiology and clinical spectrum of pediatric HAdV infections and to identify risk factors associated with hospitalization, respiratory support, and severe clinical outcomes, including the role of underlying diseases and viral coinfections.

## Material and method

### Study design and population

This retrospective observational study included children aged < 18 years who presented to Hacettepe University Children’s Hospital (Ankara, Turkey) and tested positive for HAdV by multiplex respiratory PCR between January 2014 and December 2024. Data on demographic and clinical characteristics of patients, including age, gender, underlying comorbidities, laboratory results (white blood cell count (WBC), absolute neutrophil count (ANC), absolute lymphocyte count (ALC), C-reactive protein (CRP), aspartate aminotransferase (AST), alanine aminotransferase (ALT)), diagnosis, need for respiratory support, and clinical outcomes, were extracted from electronic hospital records and evaluated retrospectively. Underlying conditions were categorized using a system-based framework rather than a single primary diagnosis. Patients could be classified across multiple clinically relevant domains, including neurologic, cardiac, pulmonary, gastrointestinal, renal, endocrine, metabolic, hematologic/oncologic, and primary immunodeficiency conditions. Immunocompromised status was further stratified according to mechanism, including hematopoietic stem cell transplantation (HSCT), solid organ transplantation (SOT), biologic therapy, systemic corticosteroid use (≥ 2 mg/kg/day), and secondary immunosuppression related to the underlying disease. Patients with missing clinical information or invalid or failed PCR results were excluded. Duplicate specimens obtained within 1 week were excluded to avoid counting the same infection episode multiple times. If the same patient tested positive again ≥ 30 days after the initial episode, accompanied by documented clinical resolution and new onset of symptoms, the episode was considered a new independent infection.

All patient records were individually reviewed, and clinical as well as laboratory findings were carefully re-evaluated to confirm the documented diagnoses. The diagnosis of sepsis was established in the presence of suspected or confirmed infection alongside a systemic inflammatory response and presence of organ dysfunction, consistent with contemporary pediatric sepsis definitions [11] For analytic purposes, a composite severe outcome was defined as pediatric intensive care unit (PICU) admission, invasive mechanical ventilation, and/or 30-day mortality, and this endpoint was used in regression analyses to evaluate predictors of severe disease among hospitalized patients. This study is in accordance with the principles of the Declaration of Helsinki and its later amendments, and was approved by the Ethics Committee of the Hacettepe University, Ankara, Turkey (SBA 25/225). Written informed consent for publication of clinical images was obtained from the parents or legal guardians of the patients.

### The detection of respiratory tract pathogens

Respiratory virus PCR testing was performed according to the clinical judgment of the treating physician in children with suspected viral infection. The clinical indications for testing included respiratory tract infection, encephalitis, fever, gastroenteritis, hepatitis, myopericarditis, sepsis, febrile seizure, conjunctivitis, hemorrhagic cystitis, and rash.

Specimens from the nasopharyngeal region of children were analyzed through multiplex RT-qPCR to detect the potential respiratory pathogens, including influenza virus, parainfluenza virus, HAdV, seasonal coronavirus, enterovirus, human rhinovirus (HRV), human bocavirus, metapneumovirus, and respiratory syncytial virus (RSV). Nucleic acid isolation was performed by Gene All Ribospin vRD II Isolation Kit, Seoul, Korea. The respiratory virus panel platform was changed on 11 May 2022 to the Ezplex Respiratory Pathogen Real-time PCR Kit. Coinfection, hospitalization, and respiratory support definitions were applied consistently across analyses. Triple viral coinfection was defined as the simultaneous detection of three distinct respiratory viral pathogens in the same nasopharyngeal PCR sample.

### Statistical analysis

The analyses were performed using the free and open-source software R (version 4.5.1, https://cran.r-project.org) and the IBM SPSS for Windows version 23.0 statistical package (Chicago, IL), with the assistance and consultation of an academic biostatistician. Normality of the data was assessed using the Kolmogorov–Smirnov test, and variance homogeneity was tested using Levene’s test. Descriptive statistics were presented as median (interquartile range (IQR)) and frequencies (percentages) as appropriate. To compare the differences between the groups, the Mann–Whitney *U* test was used for continuous variables, and Pearson chi-square test (with Yates continuity correction) were applied for categorical variables.  

Cases with HAdV and HRV codetection were excluded from the analysis, as HRV detection in respiratory samples may reflect prolonged viral shedding rather than true acute coinfection, potentially confounding the clinical attribution to HAdV. Concomitant bacterial infection was excluded as a predictor due to substantial missingness (39%, 90/231 patients) with a suspected non-random pattern, bacterial cultures and serological testing were likely ordered selectively based on clinical severity, violating the missing-at-random assumption required for valid imputation. The remaining missing data were confined to laboratory: CRP (21.2%), ALT (10.0%), AST (10.0%), absolute neutrophil count (2.6%), white blood cell count (2.2%), absolute lymphocyte count (2.2%), platelet count (2.2%). These were handled using multiple imputation by chained equations via ‘*mice*’ package. Complete clinical and demographic variables were included as auxiliary predictors in the imputation model. Candidate predictors for the multivariable model were first screened by univariable logistic regression. To identify the optimal combination of predictors, we applied automated model selection using the ‘*glmulti*’ package to reach final multivariable model. The final multivariable model was fitted using Bayesian logistic regression via ‘*arm*’ package [12–15] . 

## Results

### Demographic and clinical characteristics of inpatients and outpatients

A total of 877 HAdV infection episodes in children were included, including 601 outpatients and 276 inpatients (Table 1). The median age did not differ between groups (2 years (IQR 1–4) vs. 3 years (IQR 1–6); *p* = 0.089), and the proportion of males was similar. Underlying diseases were more common among hospitalized children (78.3% vs. 33.3%; *p* < 0.001). Outpatients had significantly higher WBC, ANC, ALC, and platelet counts than inpatients (all *p* < 0.001), while CRP levels showed no significant difference. Among inpatients, 8.6% had bacteremia, of whom 46.6% (7/15) had a central venous catheter, 2.9% had urinary tract infection as an associated diagnosis, 18.8% required PICU admission, and 32.2% required respiratory support. The median length of stay was 10 days (IQR 5–26), and 30-day mortality was 2.5%.

**Table 1:** Demographic and clinical characteristics of children with human adenovirus infection (mono-infection and viral coinfection)

	Outpatient (*n* = 601)	Inpatient (*n* = 276)	*p*-value
Age, year, median (IQR)	2 (1–4)	3 (1–6)	0.089
Male, *n* (%)	353 (59,8)	164 (59.4)	0.848
Underlying conditions, *n* (%)			
No underlying disease	471 (78.3)	92 (33.3)	< 0.001
Neurologic disease	22 (3.6)	51 (18.4)	< 0.001
Cardiac disease	16 (2.6)	40 (14.4)	< 0.001
Primary immunodeficiency	14 (2.3)	40 (14.4)	< 0.001
Renal disease	17 (2.8)	32 (11.5)	< 0.001
Gastrointestinal disease	12 (1.9)	33 (11.9)	< 0.001
Chronic lung disease	23 (3.8)	28 (10.1)	< 0.001
Allergic disease	41 (6.8)	4 (1.4)	< 0.001
Malignancy	10 (1.6)	19 (6.8)	< 0.001
Metabolic disease	9 (1.4)	24 (8.6)	< 0.001
Endocrine disease	15 (2.4)	25 (9)	< 0.001
Hematologic disease	14 (2.4)	39 (14.1)	< 0.001
Immunosuppression			
Chemotherapy	12 (1.9)	33 (11.9)	< 0.001
Biologic therapy	4 (0.6)	9 (3.2)	0.005
HSCT	5 (0.8)	21 (7.6)	< 0.001
SOT	2 (0.3)	4 (1.4)	0.082
Corticosteroid	17 (2.8)	42 (15.2)	< 0.001
Laboratory findings, median (IQR)			
WBC (× 10^9^/L)	11.8 (8.3–15.7)	9.2 (5.2–13.9)	< 0.001
ANC (× 10^9^/L)	6.7 (3.8–9.5)	4.9 (2.1–9.0)	< 0.001
ALC (× 10^9^/L)	3.2 (2.1–4.7)	2.3 (1.0–3.9)	< 0.001
Platelet (× 10^9^/L)	302 (240–382)	270 (151–381)	< 0.001
CRP (mg/L)	3.87 (1.53–9.87)	4.71 (1.29–14.70)	0.174
ALT (U/L)	15 (11–20.5)	19 (13–50)	< 0.001
AST (U/L)	33 (27–44)	37 (28–61)	< 0.001
Bacteremia, *n*/*N* (%)	-	15/174 (8.6)	NA
PICU, *n*/(%)	-	52 (18.8)	
Respiratory support, *n* (%)			NA
None	601 (100)	187 (67.8)	
O_2_ via mask	-	41 (14.9)	
NIMV	-	25 (9)	
IMV	-	23 (8.3)	
LOS, median (IQR)	-	10 (5–26)	
30-day mortality, *n*/(%)	-	7 (2.5)	NA

*WBC*, white blood cell; *ANC*, absolute neutrophil count; *ALC*, absolute lymphocyte count; *CRP*, C-reactive protein; *AST*, aspartate aminotransferase; *ALT*, alanine aminotransferase; *PICU*, pediatric intensive care unit; *LOS*, length of hospital stay; *NIMV*, noninvasive mechanical ventilation; *IMV*, invasive mechanical ventilation

A sensitivity analysis restricted to patients with HAdV mono-infection showed similar findings, with underlying diseases significantly more frequent among hospitalized children and higher WBC, ALC, and platelet counts observed in outpatients (Supplementary Table 1).

Among the seven deceased patients, six had significant underlying conditions, most commonly immunodeficiency or hemato-oncologic disorders. The predominant terminal complications were hemophagocytic lymphohistiocytosis, acute respiratory distress syndrome (ARDS), and multi-organ failure.

**Fig. 1:**
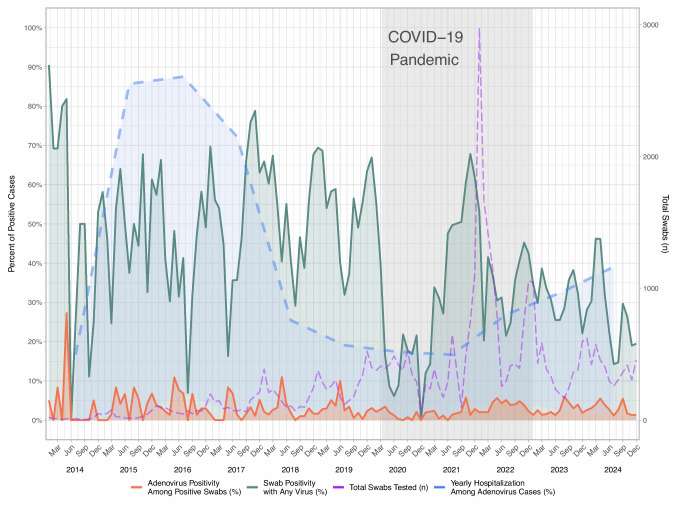
Trends in viral detection in the respiratory PCR panel and hospitalization among adenovirus-positive (HAdV) cases over time. The monthly rate of positive swab results for any respiratory pathogen (green line) and positive HAdV among PCR-positive swabs (orange line) are presented from January 2014 to December 2024. The dashed blue line represents the annual inpatient (hospitalized) rate among HAdV-positive cases. The remaining proportion represents outpatient cases. The purple dashed line indicates the monthly total number of respiratory multiplex PCR tests performed (right *y*-axis). The light-blue-shaded area indicates the gap between monthly HAdV positivity and yearly hospitalization percentage. The grey area demonstrates the COVID-19 pandemic period (March 2020 to June 2022)

Figure 1 shows the annual distribution of cases with HAdV for both inpatients and outpatients. The case numbers increased steadily from 2014 to 2019, declined in 2020, and reached a marked peak in the last quarter of 2021. Similar temporal patterns were observed for both hospitalized and non-hospitalized patients. The case numbers decreased in 2023 but remained higher than in earlier years, with a slight increase again in 2024.

**Fig. 2:**
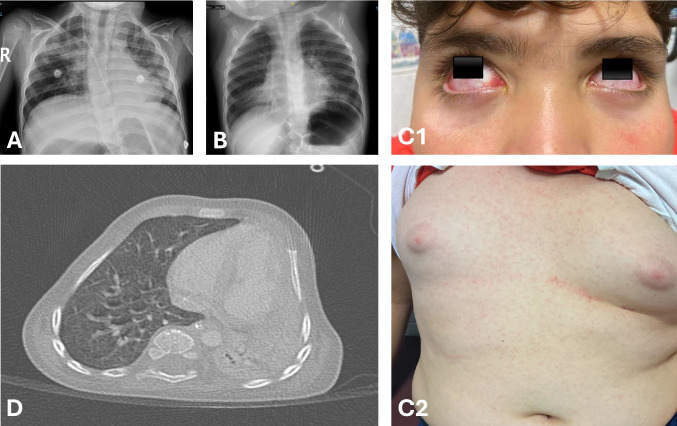
Multisystem involvement in a pediatric patient with adenovirus infection. **A** Chest X-ray demonstrating perihilar and basal infiltrates consistent with pneumonia. **B** Chest X-ray shows diffuse pulmonary opacities consistent with pneumonia. **C1** Bilateral conjunctival injection. **C2** Maculopapular rash on the trunk of the same patient as in **C1**, who was hospitalized and evaluated for multisystem inflammatory syndrome in children (MIS-C) in the differential diagnosis. **D** Thoracic computed tomography image demonstrating the right lung extending posterior to the sternum toward the left hemithorax, with marked volume loss and complete atelectasis of the lung

Images from a patient suffering from multisystem involvement in children with HAdV infection, including conjunctivitis, rash, and pulmonary infiltrates, are shown in Fig. 2.

### Comparison of the groups according to disease severity

Among all patients, 773 (88.1%) have non-severe disease and 104 (11.9%) have severe disease (Table 2). The median age was similar between the groups (3 years (IQR 1–4) in the non-severe group vs. 2 years (IQR 0–6) in the severe group, *p* = 0.3), and the proportion of males did not differ significantly (58% vs. 61.8%, *p* = 0.2).

**Table 2:** Demographic and clinical characteristics of patients with human adenovirus according to disease severity

	Non-severe (*n* = 773)	Severe (*n* = 104)	*p*-value
Age (year), median (IQR)	3 (1–4)	2 (0–6)	0.3
Male, *n* (%)	450 (58)	55 (61.8)	0.2
Underlying conditions, *n* (%)			
No underlying disease	540 (69.8)	23 (22.1)	< 0.001
Neurologic disease	45 (5.4)	28 (26.9)	< 0.001
Cardiac disease	31 (4)	25 (24)	< 0.001
Primary immunodeficiency	36 (4.6)	18 (17.3)	< 0.001
Renal disease	34 (4.3)	15 (13.4)	< 0.001
Gastrointestinal disease	24 (3.1)	21 (20.1)	< 0.001
Chronic lung disease	33 (4.2)	18 (17.3)	< 0.001
Allergic disease	42 (5.4)	3 (2.8)	0.3
Malignancy	28 (3.6)	1 (0.9)	0.2
Metabolic disease	19 (2.4)	14 (13.4)	< 0.001
Endocrine disease	27 (3.4)	13 (12.5)	< 0.001
Hematologic disease	35 (4.5)	18 (17.3)	< 0.001
Immunosuppression			
Chemotherapy	38 (4.9)	7 (6.7)	0.4
Biologic therapy	10 (1.2)	3 (2.8)	0.2
HSCT	14 (1.8)	12 (11.5)	< 0.001
SOT	3 (0.4)	3 (2.8)	0.025
Corticosteroid	0	16 (15.3)	< 0.001
Laboratory findings, median (IQR)			
WBC (× 10^9^/L)	11 (8–15)	9 (6–14)	0.035
ANC (× 10^9^/L)	6.1 (3.2–9.3)	5.1 (2.6–9.4)	0.4
ALC (× 10^9^/L)	3 (1.8–4.5)	2 (1–3.9)	< 0.001
Platelet (× 10^9^/L)	291 (225–376)	265 (119–398)	0.032
CRP (mg/L)	4 (1–11)	3 (1–15)	0.7
ALT (U/L)	16 (12–23)	22 (15–61)	< 0.001
AST (U/L)	33 (27–45)	44 (30–72)	< 0.001
Viral coinfection, *n* (%)	269 (35)	45 (43)	0.091
Bacteremia, *n*/*N* (%)	6/197 (3)	9/56 (16)	0.0003
LOS, median (IQR)	7 (3–16)	20 (9–48)	< 0.001

*WBC*, white blood cell; *ANC*, absolute neutrophil count; *ALC*, absolute lymphocyte count; *CRP*, C-reactive protein; *AST*, aspartate aminotransferase; *ALT*, alanine aminotransferase; *LOS*, length of hospital stay

Underlying medical conditions were significantly more frequent among children with severe disease. Neurologic disorders (26.9% vs. 5.4%), cardiac disease (24% vs. 4%), primary immunodeficiency (17.3% vs. 4.6%), renal disease (13.4% vs. 4.3%), gastrointestinal disease (20.1% vs. 3.1%), pulmonary disease (17.3% vs. 4.2%), metabolic disease (13.4% vs. 2.4%), endocrine disease (12.5% vs. 3.4%), and hematologic disease (17.3% vs. 4.5%) were all significantly more common in the severe group (all *p* < 0.001). In contrast, the proportion of patients without underlying disease was higher in the non-severe group (69.8% vs. 22.1%, *p* < 0.001). In our cohort, the most frequent neurologic comorbidities were epilepsy and global developmental delay, while the most common cardiac conditions included congenital heart diseases such as ASD/PFO, tetralogy of Fallot, and cardiomyopathies. Laboratory findings also differed between groups. Children with severe disease had lower white blood cell counts (9 vs. 11 × 10^9^/L, *p* = 0.035), lower lymphocyte counts (2 vs. 3 × 10^9^/L, *p* < 0.001), and lower platelet counts (265 vs. 291 × 10^9^/L, *p* = 0.032), while AST and ALT levels were significantly higher in the severe group (AST 44 vs. 33 U/L; ALT 22 vs. 16 U/L; both *p* < 0.001). Viral coinfection rates did not differ significantly between the groups (43% vs. 35%, *p* = 0.091). However, bacteremia was more common among patients with severe disease (16% vs. 3%, *p* < 0.001).

### Comparison of cases according to the status of mono-infection and viral coinfection

Of the 877 episodes, 563 (64.2%) had HAdV as mono-infection, whereas 314 (35.8%) show coinfections (Table 3). The median age did not differ between the groups (3 vs. 2 years; *p* = 0.454), and the proportion of males was similar.

**Table 3:** Demographic and clinical characteristics of patients with human adenovirus according to mono-infection and viral coinfection

	Mono-infection (*n* = 563)	Coinfection (*n* = 314)	*p*-value
Age, year, median (IQR)	3 (1–5)	2 (1–4)	0.454
Male, *n* (%)	331 (58.8)	186 (59.2)	0.898
Underlying conditions, *n* (%)			
No underlying disease	376 (66.8)	187 (59.6)	0.032
Neurologic disease	42 (7.5)	31 (9.9)	0.2
Cardiac disease	37 (6.6)	19 (6.1)	0.8
Primary immunodeficiency	26 (4.6)	28 (8.9)	0.011
Renal disease	27 (4.8)	22 (7)	0.2
Gastrointestinal disease	20 (3.6)	25 (8)	0.005
Chronic lung disease	30 (5.3)	21 (6.7)	0.4
Allergic disease	26 (4.6)	19 (6.1)	0.4
Malignancy	23 (4.1)	6 (1.9)	0.083
Metabolic disease	12 (2.1)	21 (6.7)	< 0.001
Endocrine disease	21 (3.7)	19 (6.1)	0.12
Hematologic disease	33 (5.9)	20 (6.4)	0.8
Immunosuppression			
Chemotherapy	31 (5.5)	14 (4.5)	0.5
Biologic therapy	7 (1.2)	6 (1.9)	0.6
HSCT	12 (2.1)	14 (4.5)	0.052
SOT	5 (0.9)	1 (0.3)	0.4
Corticosteroid	37 (6.6)	22 (7)	0.8
Hospitalization, *n* (%)			0.123
Inpatient	167 (29.7)	109 (34.7)	
Outpatient	396 (70.3)	205 (65.3)	
Laboratory findings, median (IQR)			
WBC (× 10^9^/L)	11 (7.7–15)	10.6 (5.9–14.7)	0.144
ANC (× 10^9^/L)	6.1 (3.3–9.3)	5.3 (2.5–9)	0.116
ALC (× 10^9^/L)	3 (1.7–4.3)	2.6 (1.5–4.4)	0.256
Platelet (× 10^9^/L)	288 (217–381)	285 (209–380)	0.591
CRP (mg/L)	4.4 (1.6–13.9))	3.5 (1.1–7.9)	0.029
ALT (U/L)	16 (12–26)	17 (13–35)	0.137
AST (U/L)	33 (27–47)	36 (28–49)	0.066
Bacteremia, *n*/*N* (%)	8/186 (4.3)	7/84 (8.3)	0.181
PICU (*n* %)	33 (5.9)	19 (6.1)	> 0.05
Respiratory support, *n* (%)			0.103
None	513 (91.1)	275 (87.6)	
O_2_ via mask	23 (4.1)	18 (5.7)	
NIMV	11 (2)	14 (4.5)	
IMV	16 (2.8)	7 (2.2)	
LOS (day)	9 (5–24)	10 (5–32)	0.593
30-day mortality (*n* %)	4 (0.7)	3 (0.9)	0.706

*WBC*, white blood cell count; *ANC*, absolute neutrophil count; *ALC*, absolute lymphocyte count; *CRP*, C-reactive protein; *AST*, aspartate aminotransferase; *ALT*, alanine aminotransferase; *PID*, primary immunodeficiency; *HSCT*, hematopoietic stem cell transplantation; *SOT*, solid organ transplantation; *PICU*, pediatric intensive care unit; *LOS*, length of hospital stay; *NIMV*, noninvasive mechanical ventilation; *IMV*, invasive mechanical ventilation

An underlying disease was significantly more common in the coinfection group than in the mono-infection group (*p* = 0.032). The laboratory findings, including WBC, ANC, ALC, platelet count, ALT, and AST, were similar between groups. CRP levels were higher in the mono-infection group (*p* = 0.029). Bacteremia rates (4.3% vs. 8.3%; *p* = 0.181), PICU admission (5.9% vs. 6.1%; *p* > 0.05), respiratory support (8.9% vs. 12.4%; *p* = 0.103), and length of stay (9 vs. 10 days; *p* = 0.593) demonstrated no significant differences. Thirty-day mortality was low and comparable (0.7% vs. 0.9%; *p* = 0.706).

Further analysis of viral coinfection patterns demonstrated that HRV (15.1%) was the most common copathogen, whereas other respiratory viruses such as seasonal coronaviruses (3.7%), bocavirus (3.5%), influenza (3.2%), parainfluenza (2.1%), and RSV (1.8%) are detected less frequently (Supplementary Table 2).

### Risk factors associated with hospitalization and severe outcome

The multivariable analysis shows several factors associated with severe outcomes (Table 4). Male sex (OR 2.30, 95% CI 1.23–4.29; *p* = 0.009), neurologic disease (OR 2.12, 95% CI 1.01–4.42; *p* = 0.045), cardiac disease (OR 3.27, 95% CI 1.49–7.17; *p* = 0.003), and a history of HSCT (OR 4.32, 95% CI 1.36–13.7; *p* = 0.013) were independently associated with increased risk of severe outcomes.

**Table 4:** Risk factors associated with hospitalization and severe outcomes in patients with adenovirus infection*

Variables	Univariate OR (95% CI)/*p*-value	Multivariate OR (95% CI)/*p*-value
Composite severe outcome		
Male	1.98 (1.13–3.52)/0.018	2.30 (1.23–4.29)/**0.009**
ANC	1.06 (1.00–1.12)/0.058	1.05 (0.98–1.12)/0.2
Neurologic condition	2.20 (1.12–4.36)/0.022	2.12 (1.01–4.42)/**0.045**
Cardiac condition	3.29 (1.60–7.00)/0.001	3.27 (1.49–7.17)/**0.003**
Pulmonary condition	2.68 (1.14–6.51)/0.025	2.26 (0.91–5.59)/0.076
Allergic condition	5.30 (0.67–108)/0.2	5.34 (0.71–40.1)/0.10
Endocrinologic condition	2.30 (0.87–6.26)/0.093	2.12 (0.75–5.99)/0.2
Chemotherapy-related immunosuppression	0.26 (0.08–0.72)/0.017	0.40 (0.12–1.29)/0.12
HSCT	2.65 (0.98–7.56)/0.058	4.32 (1.36–13.7)/**0.013**
SOT	5.30 (0.67–108)/0.2	3.50 (0.50–24.4)/0.2
Hospitalization		
ALC	1.09 (1.02–1.17) 0.018	0.96 (0.90–1.02) 0.2
CRP	0.99 (0.98–1.00) < 0.001	1.01 (1.00–1.01) 0.085
ALT	1.00 (1.00–1.00) 0.002	1.00 (1.00–1.00) 0.13
No underlying disease	7.16 (5.09–10.2)	0.14 (0.09–0.22) < **0.001**
Neurologic condition	0.14 (0.08–0.26) < 0.001	1.86 (0.95–3.61) 0.068
PID	0.12 (0.05–0.296) < 0.001	2.05 (0.88–4.79) 0.10
Allergic condition	4.03 (1.58–13.6) 0.009	0.08 (0.03–0.22) < **0.001**
Endocrinologic condition	0.26 (0.12–0.55) < 0.001	0.56 (0.23–1.35) 0.2
HSCT	0.10 (0.03–0.27) < 0.001	2.74 (0.86–8.70) 0.087

*ALC*, absolute lymphocyte count; *CRP*, C-reactive protein; *AST*, aspartate aminotransferase; *ALT*, alanine aminotransferase; *HSCT*, hematopoietic stem cell transplantation; *SOT*, solid organ transplantation; *PID*, primary immunodeficiency; *OR*, odds ratio; *CI*, confidence interval

^*^In the sensitivity analysis, patients with rhinovirus co-detection or bacteremia were excluded

For hospitalization, multivariable analysis showed that the absence of underlying disease was associated with a lower risk of hospitalization (OR 0.14, 95% CI 0.09–0.22; *p* < 0.001), whereas allergic conditions were associated with reduced hospitalization risk (OR 0.08, 95% CI 0.03–0.22; *p* < 0.001). Other variables were not independently associated with hospitalization in the adjusted model.

## Discussion

In this large cohort of children with HAdV, a substantial proportion required hospital-based care. Approximately one-third of patients required hospitalization, highlighting the considerable clinical burden associated with HAdV infection in the pediatric population. In addition, nearly one in nine patients experienced a severe outcome, defined as the need for PICU admission, invasive mechanical ventilation, or death within 30 days of diagnosis. These findings indicate that, although HAdV infection is often considered a self-limited illness in children, however, a meaningful number of patients may develop severe disease requiring advanced supportive care.

Several host-related factors were associated with a severe clinical outcome in our cohort. Multivariable analysis demonstrated that male sex, neurologic disorders, congenital cardiac disease, and HSCT were independent predictors of severe outcomes. These findings align with prior pediatric and adult data demonstrating that chronic medical conditions independently increase the risk of severe HAdV respiratory disease [4, 16]. Congenital heart disease has been associated with increased morbidity and mortality in viral lower respiratory tract infections, including HAdV [5]. Similarly, children with neurological disorders appear particularly vulnerable due to impaired airway protection, limited physiological reserve, and dysregulated inflammatory responses [17]. The higher requirement for respiratory support and intensive care in this subgroup further supports their increased susceptibility to severe clinical outcomes [18]. Furthermore, children with a history of HSCT represent a particularly high-risk group due to profound immune dysregulation and impaired viral clearance [19, 20].

In addition to these clinical severity indicators, inflammatory markers were also evaluated. Although CRP levels were higher in the aggregated mono-infection group, stratified analysis demonstrated considerable variability across specific viral combinations, suggesting that coinfection does not represent a uniform inflammatory phenotype. Consistent with this, a recent retrospective cohort study by Strempas et al. [21] reported that detection of multiple respiratory pathogens was generally not associated with higher CRP levels compared with mono-infection, except for one specific viral pairing (parainfluenza virus with RSV). These findings indicate that the inflammatory response is likely pathogen-specific rather than determined by the number of detected viruses.

Beyond respiratory disease, HAdV also exhibits broad tissue tropism and is capable of causing clinically significant extrapulmonary manifestations [4]. In our cohort, a subset of children presented with extrapulmonary syndromes, most notably gastroenteritis, consistent with previous epidemiologic studies [22, 23]. Large multicenter data have similarly shown that certain HAdV species are associated with non-respiratory manifestations, supporting the concept of type-specific clinical patterns [22]. Beyond gastrointestinal involvement, HAdV has been associated with neurologic and cardiac complications, including encephalitis and myocarditis, which may result in significant morbidity [4]. In our cohort, severe extrapulmonary manifestations including sepsis, meningitis, and encephalitis accounted for 2.5% of initial presentations.

The frequency of sepsis in our hospitalized cohort is consistent with prior evidence that HAdV may precipitate systemic inflammatory dysregulation, particularly in young or medically complex children [24, 25]. Given the retrospective design, the relative contribution of viral versus bacterial pathogens cannot be definitively established. Recent evidence suggests that adenovirus-associated sepsis is characterized by persistent adenoviremia and dysregulated host responses leading to life-threatening organ dysfunction, in line with contemporary sepsis definitions. We observed that elevated serum viral load and disseminated viremia have been associated with increased mortality, supporting the hypothesis that HAdV may act as a primary source of sepsis rather than a secondary respiratory pathogen [4]. Although causality cannot be conclusively approved in this retrospective analysis, the clinical patterns observed in our cohort together with emerging mechanistic data may support a plausible pathogenic role of HAdV in the development of sepsis in a subset of patients.

In our cohort, seven children died, nearly all with significant underlying medical conditions. This observation is consistent with prior reports demonstrating that children with complex chronic illnesses, particularly those with immunodeficiency or hemato-oncologic disorders, are at markedly increased risk for severe and fatal HAdV infection. Impaired viral clearance and dysregulated host immune responses likely contribute to this increased vulnerability [20].

The terminal clinical features, including HLH, ARDS, and multi-organ failure, reflect the profound immune dysregulation that HAdV can precipitate in high-risk hosts. Similar patterns have been described in previous studies, supporting the hypothesis that HAdV, although classically regarded as a respiratory pathogen, may function as a systemic inflammatory driver in selected patients [26].

Viral coinfection was observed in more than one-third of patients in our cohort, reflecting the high frequency of multiple respiratory virus detection in children with HAdV infection. However, the presence of viral coinfection was not associated with increased hospitalization, need for respiratory support, intensive care admission, or mortality. These findings may suggest that the clinical course of HAdV infection may be primarily driven by host-related factors rather than the presence of additional respiratory viruses [27]. Similar observations have been reported in previous studies of pediatric respiratory infections, where viral codetection did not consistently correlate with disease severity [28, 29].

Despite these strengths of our data, several limitations should be acknowledged. The absence of adenovirus type analysis represents an important limitation of the present study. Possibly, the observed severity patterns or the marked increase in cases in the last quarter of 2021 may have been influenced by shifts in circulating genotypes, which cannot be confirmed due to the origin of the study. Future prospective surveillance including genotype analysis and viral load assessment would be relevant to better demonstrate the relationship between HAdV type and clinical severity.

Furthermore, the retrospective study design may influence the completeness and uniformity of the clinical data, particularly regarding symptom onset and timing of sample collection. Furthermore, the absence of a non-HAdV control group precludes a definitive assessment of mortality attributable specifically to HAdV infection. Lastly, the study was performed in a single tertiary-care center, and thus, the findings may not be fully generalizable to other regions or healthcare settings with differing epidemiologic patterns, diagnostic capacities, or patient populations.

In this cohort of children with laboratory-confirmed HAdV infection, underlying medical conditions, particularly neurometabolic and congenital cardiac disorders, were associated with an increased risk of hospitalization and severe clinical outcomes. HAdV demonstrated a broad clinical spectrum extending beyond respiratory disease, including extrapulmonary manifestations such as gastroenteritis, sepsis, and neurologic or cardiac complications. Although viral coinfections were frequent, they were not associated with increased disease severity in our population. These findings underscore the clinical heterogeneity of pediatric HAdV infection.

## Supplementary Information

Below is the link to the electronic supplementary material.

ESM 1 (DOCX 23.9 KB)

ESM 2 (DOCX 39.5 KB)

## Data Availability

The data generated during the current study are available from the corresponding authors on a reasonable request.

## Declarations

### Use of large language models (LLMs)

AI-assisted copyediting was used to improve the readability, grammar, and style of the manuscript text. All AI-assisted content was carefully reviewed, edited, and approved by the authors to ensure accuracy and accountability.
