# Supplementary material for: Pediatric adenovirus infections: 10-year clinical spectrum and predictors of severe disease with emphasis on comorbidities and coinfections
Source: Eur J Pediatr. 2026 Apr 24;185(5):296. doi: 10.1007/s00431-026-06952-0 (PMC13109187; doi:10.1007/s00431-026-06952-0)
Supplement: Supplementary file 1 — (DOCX 23.9 KB) [file 431_2026_6952_MOESM1_ESM.docx]

| Supplementary Table 1. Clinical and laboratory characteristics of hospitalized and outpatient children with adenovirus monoinfection | | | |
| --- | --- | --- | --- |
|  | Inpatient  (*n*=167) | Outpatient  (*n*=396) | *p*-value |
| Age, year, median (IQR) | 3 (1 – 6) | 3 (1 – 4) | 0.3 |
| Male, n (%) | 99 (59.3) | 232 (58.6) | 0.9 |
| Underlying conditions, n (%)  No underlying disease  Neurologic disease  Cardiac disease  Primary immunodeficiency  Renal disease  Gastrointestinal disease  Chronic lung disease  Allergic disease  Malignancy  Metabolic disease  Endocrine disease  Hematologic disease  Immunosuppression  Chemotherapy  Biologic therapy  HSCT  SOT  Corticosteroid | 60 (35.9)  30 (18)  28 (16.8)  19 (11.4)  16 (9.6)  12 (7.2)  16 (9.6)  2 (1.2)  15 (9)  9 (5.4)  12 (7.2)  26 (15.6)  23 (13.8)  6 (3.6)  10 (6)  4 (2.4)  26 (16.5) | 316 (79.8)  12 (3)  9 (2.3)  7 (1.8)  11 (2.8)  8 (2)  14 (3.5)  24 (6.1)  8 (2)  3 (0.8)  9 (12.3)  7 (1.8)  8 (2)  1 (0.3)  2 (0.5)  1 (0.3)  11 (2.8) | <0.001  <0.001  <0.001  <0.001  <0.001  0.002  0.004  0.012  <0.001  0.001  0.005  <0.001  <0.001  0.003  <0.001  0.029  <0.001 |
| Laboratory findings,  median (IQR)  WBC  ANC  ALC  Platelet  CRP  ALT  AST | 9.4 (5.7 – 14.)  5.6 (2.5 – 9.3)  2.3 (1.26 – 3.94)  265 (144 – 379)  6 (1 – 16)  19 (13 – 39)  36 (28 – 55) | 11.8 (8.7 – 16)  6.7 (4.2 – 9.4)  3.26 (2.25 – 4.95)  305 (249 – 385)  4 (2 – 13)  15 (11 – 21)  33 (26 – 44) | <0.001  <0.001  <0.001  <0.001  0.174  <0.001  <0.001 |
| Bacteremia, n/N (%) | 8/105 (7.6) | - | NA |
| PICU, n/(%) | 33 (19.8) | - | NA |
| Respiratory support, n (%)  None  O_2_ via mask  NIMV  IMV | 117 (70.1)  23 (13.8)  11 (6.6)  16 (9.6) | 396 (100) | NA |
| LOS, median (IQR) | 9 (5-23) | - | NA |
| 30-day mortality, n/(%) | 4 (2.4) | - | NA |
| WBC: White blood cell; ANC: Absolute neutrophil count; ALC: Absolute lymphocyte count; CRP: C-reactive protein; AST: Aspartate aminotransferase; ALT: Alanine aminotransferase; PICU: Pediatric intensive care unit; LOS: Length of hospital stay. Others^#^: genetic, endocrinologic, autoimmune disorders, prematurity, NIMV: Noninvasive mechanical ventilation, IMV:Invasive mechanical ventilation | | | |
