# Supplementary material for: Pediatric adenovirus infections: 10-year clinical spectrum and predictors of severe disease with emphasis on comorbidities and coinfections
Source: Eur J Pediatr. 2026 Apr 24;185(5):296. doi: 10.1007/s00431-026-06952-0 (PMC13109187; doi:10.1007/s00431-026-06952-0)
Supplement: Supplementary file 2 — (DOCX 39.5 KB) [file 431_2026_6952_MOESM2_ESM.docx]

| Supplementary Table 2. Demographic and clinical characteristics of patients with human adenovirus mono and co-infection with each respiratory virus. | | | | | | | | |
| --- | --- | --- | --- | --- | --- | --- | --- | --- |
|  | **HAdV**  (n=563) | **HAdV + HBoV** (n=32) | **HAdV + HRV** (n=133) | **HAdV + IFV** (n=28) | **HAdV + PIV**  (n=19) | **HAdV + RSV** (n=16) | **HAdV + sCoV** (n=33) | **Triple viruses** (n=48) |
| Age, year*^1^* | 3 (1 – 5) | 1 (1 – 4) | 2 (1 – 5) | 4 (2 – 6) | 1 (0 – 3) | 1.5 (0 – 3) | 2 (1 – 4) | 3 (1 – 5) |
| Male, (n %) | 331 (58.8) | 16 (50) | 78 (58.6) | 16 (57.1) | 14 (74) | 9 (56.3) | 21 (63.6) | 31 (62) |
| Underlying conditions, n (%)  No underlying disease  Neurologic disease  Cardiac disease  PID  Renal disease  Gastrointestinal disease  Chronic lung disease  Allergic disease  Malignancy  Metabolic disease  Endocrine disease  Hematologic disease  Immunosuppression  Chemotherapy  Biologic therapy  HSCT  SOT  Corticosteroid | 376 (67)  42 (7)  37 (7)  26 (5)  27 (5)  20 (4)  30 (5)  26 (5)  23 (4)  12 (2)  21 (4)  33 (6)  31 (6)  7 (1)  12 (2)  5 (1)  37 (7) | 22 (32)  2 (6)  2 (6)  0  1 (3)  3 (9)  2 (16)  1 (3)  0  4 (13)  3 (9)  3 (9)  0  0  1 (3)  0  1 (3) | 77 (58)  15 (11)  6 (5)  16 (12)  13 (10)  13 (10)  9 (7)  7 (5)  3 (2)  12 (9)  11 (8)  8 (6)  7 (5)  4 (3)  5 (4)  1 (1)  11 (8) | 19 (64.3)  3 (11)  2 (7))  2 (7)  0  2 (7)  2 (7)  3 (11)  1 (4)  2 (7)  0  0  1 (4)  0  1 (4)  0  1 (4) | 7 (37)  3 (16)  4 (21)  0  2 (11)  1 (5)  1 (5)  3 (16)  0  1 (5)  0  0  0  1 (5)  0  0  1 (5) | 12 (75)  1 (6)  1 (6)  3 (19)  0  1 (6)  0  0  0  1 (6)  1 (6)  2 (13)  0  0  0  0  1 (6) | 18 (55)  3 (9)  3 (9)  3 (9)  4 (12)  1 (3)  2 (6)  2 (6)  0  0  1 (3)  4 (12)  3 (9)  1 (3)  1 (3)  0  3 (9) | 29 (60)  4 (8)  1 (2)  4 (8)  2 (4)  4 (8)  4 (8  3 (6)  2 (4)  1 (2)  2 (4)  3 (6)  3. (6)  0  6 (13)  0  4 (8) |
| Hospitalization, n (%)  Inpatient  Outpatient | 167 (29.7)  396 (70.3) | 12 (37.5)  20 (62.5) | 45 (33.8)  88 (66.2) | 8 (28.6)  20 (71.4) | 7 (36.8)  12 (63.1) | 6 (37.5)  10 (62.5) | 12 (36.4)  21 (63.6) | 17 (35)  31 (65) |
| Diagnosis, (n %)      RTI  Encephalitis      Fever      Gastroenteritis      Hepatitis      Myopericarditis      Others*      Sepsis | 374 (66.6)  2 (0.4)  75 (13.3)  40 (7.1)  6 (1.1)  3 (0.5)  54 (9.6)  9 (1.6) | 24 (75)  0  4 (12.5)  2 (6.3)  0  0  2 (6.3)  0 | 100 (75.2)  0  15 (11.3)  5 (3.8)  1 (0.8)  2 (1.5)  7 (5.3)  3 (2.3) | 21 (75)  0  4 (14.3)  2 (7.1)  0  0  1 (3.6)  0 | 16 (84.2)  0  2 (10.5)  0  0  0  1 (5.2)  0 | 12 (75)  0  1 (6.3)  0  0  0  3 (18.8)  0 | 20 (60.6)  0  6 (18.2)  5 (15.2)  1 (3)  0  1 (3)  0 | 28 (58)  0  10 (21)  2 (4)  1 (2)  0  6 (13)  1 (2) |
| Laboratory findings  WBC (×10⁹/L)  ANC (×10⁹/L)  ALC (×10⁹/L)  Platelet (×10⁹/L)  CRP (mg/L)  ALT (U/L)  AST (U/L) | 11 (7.7 – 15)  6.1 (3.3 – 9.3)  3 (1.7 – 4.3)  287 (216 – 381)  4.4 (1.6 – 13.9)  16 (12 – 26)  33 (27 – 47) | 12.1 (8.4 – 14.8)  6.8 (4 – 9)  3.4 (2.3 – 4.8)  329 (248 – 372)  4.6 (2.2 – 7.3)  15.5 (14 – 44.5)  39.5 (31.5 – 56.5) | 10 (6 – 14)  5.3 (2.6 – 9.4)  2.5 (1.5 – 4.6)  234 (209 – 394)  3.2 (1.2 – 9.5)  16 (12 – 22)  33 (27 – 46) | 5.2 (3 – 8.9)  2.6 (1 – 5.4)  1.6 (1.3 – 2.1)  219 (166 – 252)  1.9 (0.5 – 4.4)  16 (11 – 19)  36 (24 – 44) | 9.3 (8.6 – 14.9)  4.5 (3 – 10.1)  3.9 (2.8 – 4.9)  278 (238 – 311)  5.6 (0.6 – 7.7)  16 (8 – 19)  29 (25 – 66) | 11.2 (10 – 13.1)  6 (2.4 – 9.5)  3.7 (1.5 – 5.3)  338 (244 – 415)  4 (2.2 – 5.7)  22 (9.5 – 151.5)  34 (28.5 – 189.5) | 13.4 (5.4 – 15.1)  7 (3.1 – 9.6)  2.9 (1.9 – 5.3)  365 (143 – 511)  5.9 (1.1 – 25.9)  18 (14 – 47)  37 (33 – 49) | 9.4 (4.1 – 15.2)  5.3 (2.4 – 11.1)  2.2 (1.2 – 3.3)  265 (199 – 351)  3 (0.8 – 10.1)  20 (13.2 – 64.5)  43.5 (30.2 – 67.5) |
| Bacteremia, (n %) | 8/186 (4.3) | 0/8 (0) | 4/33 (12.1) | 0/10 (0) | 1/4 (25) | 0/3 (0) | 1/10 (10) | 1/13 (8) |
| ICU, (n %) | 33 (5.9) | 4 (12.5) | 6 (4.5) | 1 (3.6) | 1 (5) | 1 (6.3) | 2 (6.1) | 4 (8) |
| Respiratory support,  (n %) | 50 (8.9) | 3 (9.4) | 18 (13.5) | 2 (7.1) | 3 (16) | 2 (12.5) | 4 (12.1) | 7 (14) |
| Oxygen | 20 (3.5) | 2 (6.2) | 8 (6) | 0 | 2 (11.7) | 0 | 2 (6) | 4 (8) |
| NIMV | 14 (2.5) | 0 | 7 (5.2) | 2 (7.1) | 1 (5.8) | 1 (6.2) | 0 | 3 (6) |
| IMV | 16 (2.8) | 1 (3.1) | 3 (2.2) | 0 | 0 | 1 (6.2) | 2 (6) | 0 |
| LO | 9 (5 – 23) | 8 (1 – 14) | 11 (5 – 45) | 10 (2 – 23) | 5 (3 – 22) | 12 (7 – 29) | 18 (7 – 44) | 18 (9– 32) |
| Infection related mortality, (n %) | 4 (0.7) | 0 | 2 (1.5) | 0 | 0 | 1 (6.3) | 0 | 0 |
| *^1^*Data are shown as n/N (%) or median (IQR), MPV (n=3) and SARS-CoV-2 (n=2) were excluded due to low case numbers, PID: Primary immunodeficiency; RTI: Respiratory tract infection; UTI: Urinary tract infection; WBC: White blood cell; ANC: Absolute neutrophil count; ALC: Absolute lymphocyte count; CRP: C-reactive protein; AST: Aspartate aminotransferase; ALT: Alanine aminotransferase; ICU: Intensive care unit; LOS: Length of hospital stay. Others included febrile seizure, conjunctivitis, hemorrhagic cystitis, rash, and fever of unknown origin. | | | | | | | | |
